# Reexamination of diathesis stress and neurotoxic stress theories: A qualitative review of pre‐trauma neurobiology in relation to posttraumatic stress symptoms

**DOI:** 10.1002/mpr.1864

**Published:** 2020-11-21

**Authors:** Michael S. Scheeringa

**Affiliations:** ^1^ Department of Psychiatry and Behavioral Sciences Tulane University School of Medicine New Orleans Louisiana USA

**Keywords:** diathesis stress, neurobiology, posttraumatic stress disorder, qualitative review, toxic stress

## Abstract

**Objective:**

Associations of neurobiological differences with posttraumatic stress disorder (PTSD) have generated interest in their temporal relation. Support has been voiced for the neurotoxic stress theory (NST) in which neurobiological differences develop following exposure and PTSD development. In contrast, the diathesis stress theory (DST) posits that neurobiological differences existed prior to exposure and may be vulnerability factors for PTSD. Studies in the first wave of neurobiological PTSD research were all cross sectional, but a second wave of research followed which used prospective repeated‐measures designs that measured neurobiology prior to trauma exposure experiences, allowing greater causal inference.

**Methods:**

This study reviewed the second‐wave studies in hopes of developing a preliminary consensus to support either the NST or the DST based on this more powerful prospective, repeated‐measures study design.

**Results:**

Twenty‐five second‐wave studies were located that measured neurobiology prior to traumatic experiences. Nineteen studies supported the DST. Of 10 studies that were capable of testing the NST, only 3 were supportive.

**Conclusion:**

The implications of the NST versus the DST have profound implications for understanding the fragility of the human brain and possible paths forward for future research on assessment, treatment, and social policy.

## 1 INTRODUCTION

### 1.1 Background

Two inferences emerge from surveying the first 35 years of research on the relation of neurobiology to posttraumatic stress disorder (PTSD). The first inference is a consensus among trauma researchers, clinicians, and stakeholders that trauma permanently alters the brain (Shonkoff, Garner, Siegel, & Wood, 2012; van der Kolk, 2014), also known as the neurotoxic stress theory (NST). The second inference is a body of evidence from pre‐trauma prospective studies that has arisen in the last 14 years which provides strong evidence that many, if not all, of the neurobiological differences that have been discovered in PTSD are preexisting vulnerability factors, and tests that were capable of detecting permanent neural alterations to the brains of trauma victims have been mostly negative (Danese et al., 2017; DiGangi et al., 2013). These two inferences are not mutually exclusive, but each implies quite different understandings of how resilient the human mind is to defend against psychological stress. The purpose of this review is to examine this body of pre‐trauma prospective studies to determine what ought to be the consensus, if any, about the causal relationship between neurobiology and PTSD.

The two main theories that can potentially explain the existence of neurobiological differences associated with PTSD are the NST and the diametrically opposing diathesis stress theory (DST). The DST attempts to explain psychopathology as the consequence of a predispositional vulnerability (the diathesis) and stressful life experiences. DST is the dominant model for explaining psychiatric disorders. The DST has been first attributed to Paul Meehl's application of the model to schizophrenia in the 1960s, and has been applied to depression, anxiety, bipolar disorder, and other disorders (Zuckerman, 1999). The DST suggests that there are individual differences in predisposing factors (usually genetic and biological) for disorders. In the context of a stressor, those with the diathesis are at higher risk of developing the disorder. Applied to PTSD, the DST would suggest that individuals with a preexisting neurobiological vulnerability are at higher risk for the development of PTSD symptoms following the psychological distress resulting from trauma.

The NST posits that the organisms' brains were functionally normal prior to insult, and then the introduction of toxic agents either temporarily or permanently altered one or more functional capacities (Shonkoff et al., 2012). Applied to PTSD, the NST would suggest that rather than preexisting neurobiological differences between those who do and do not develop PTSD, the psychological distress resulting from trauma causes neural alterations, which then leads to the development of PTSD symptoms. Toxic agent theories historically have been more useful for explaining medical diseases due to living pathogens (e.g., bacterial infections), ingested chemicals (e.g., lead or alcohol), or airborne particles (e.g., asbestos fibers). The NST is unique for positing psychological distress as the instigating toxic agent, which triggers an endogenous chemical that rises to toxic levels. Variations of the NST theory are also known as toxic stress (Shonkoff, Boyce, & McEwen, 2009), biological embedding (Danese, 2018), or how experience “gets under the skin” (McEwen, 2012).

Each theory has profoundly different implications for understanding the nature of psychopathology in humans. The purpose of this review is to determine the extent of scientific evidence in humans that exists for each theory from prospective, repeated‐measures studies.

### 1.2 First wave of PTSD neurobiology research, 1986–2005

A first wave of research on the association of neurobiology with PTSD may be characterized as having been conducted on humans between 1986 and 2005 when the study designs were exclusively cross sectional. Pioneering studies began with cortisol (Mason, Giller, Kosten, Ostroff, & Podd, 1986) and heart rate (Pallmeyer, Blanchard, & Kolb, 1986) in 1986, and then brain imaging in 1995 (Bremner et al., 1995). Based on this first wave of research, evidence indicated that individuals with PTSD were different on a number of neurobiological indices compared to individuals who had not developed PTSD, including volume of brain structures, activation of brain structures, peripheral autonomic nervous system, and hypothalamic–pituitary–adrenal (HPA) axis indices for both resting and reactivity, though directionality of differences was oftentimes inconsistent. Despite these advances, the major limitation of the first wave of research is that the studies were cross‐sectional designs, views taken as single snapshots in time, which cannot take advantage of the temporal sequence of events, and therefore have essentially no ability to clarify causal relationships. Cross‐sectional studies have no ability to determine whether neurobiological differences existed prior to the trauma experiences and perhaps served as vulnerability factors, or if the differences developed as consequences of trauma and were indices of permanent neural alterations to the brain.

### 1.3 Second wave, 2005 to the present

The second wave includes studies that examined subjects on at least two occasions, including measurement of neurobiological variables prior to index trauma exposures, and PTSD symptoms following exposures. These types of pre‐trauma prospective studies have the power that is absent from cross‐sectional studies of testing temporal, and perhaps causal, relationships between neurobiology and PTSD. Although pre‐trauma prospective studies are more powerful for understanding causal relationships compared to cross‐sectional studies, pre‐trauma prospective studies are extremely difficult to conduct with humans for ethical and logistical reasons.

To illustrate, the first pre‐trauma prospective study of neurobiology, conducted by Guthrie and Bryant and published in 2005, examined skin conductance (SC) during a startle response paradigm in 87 firefighter recruits prior to their first year of active duty. The researchers assessed the firefighters again after they were exposed to life‐threatening events of active duty. By knowing their SC responses prior to exposures, and measuring changes in their PTSD symptoms, they could test the DST. In addition, by measuring their SC responses a second time following exposures, they could also test the NST (Guthrie & Bryant, 2005).

### 1.4 The present study

In 2013, DiGangi and colleagues published the first systematic review of second‐wave (i.e., pre‐trauma prospective) studies that measured neurobiological or cognitive variables prior to trauma exposures (DiGangi et al., 2013). They concluded that the majority of differences in these variables predated trauma exposure, and, consistent with the DST, these differences probably served as vulnerability factors for the development of posttrauma psychological problems. DiGangi et al.‘s conclusion contravened the current consensus in favor of the NST.

The aim of this review is to determine, in light of more recent studies that have been published since the review by DiGangi et al. which measured neurobiology pre‐trauma, if there is enough evidence for a consensus about a causal relationship, if any, between psychological trauma and neurobiology.

## 2 METHODS

A systematic review was conducted in PsychInfo and PubMed consistent with the method of DiGangi et al. (2013). Following the Preferred Items for Systemic Reviews and Meta‐Analysis guidelines, the Boolean search string used was AB (PTSD or exact “posttraumatic stress disorder”) or (exact “pre‐trauma” or exact “pretrauma” or exact “pre‐combat”) and (exact “risk factor*” or exact “vulnerability factor*” or neurobiolog*) and (longitudinal or prospective). Search filters limited results to peer‐reviewed articles, human subjects, and the time period of 1980–2020. All languages were permitted. Reference lists of included articles and review articles were checked for additional studies. Inclusion criteria required that (1) a neurobiological variable was measured prior to an index trauma exposure, and (2) symptoms of PTSD were assessed at least 1 month following exposure. Symptoms could be either a dimensional measure of posttraumatic stress symptom (PTSS) severity or a categorical measure of PTSD.

Next, we made a list of all the relevant terms that were included in the abstracts of the studies that were found through the first search. These terms included trauma, stress, prospective, longitudinal, neural, biological, physiological, and 29 neurobiological terms (adrenal, glucocorticoid, hippocampal, heart rate, etc.). Next, we selected a subset of those terms that would have captured all of the studies that we had found. This resulted in a new search strategy of abstracts with 12 terms: trauma and amygdala or autonomic or catecholamine or conductance or cortex or cortisol or electromyogram or genes or glucocorticoid or hippocampal or psychophysiological, and limited to prospective studies.

Evidence for NST was considered present if a neurobiological variable that had been measured prior to index traumas changed following traumas, and the change associated significantly with PTSS or PTSD. Evidence for DST was considered present if a neurobiological variable that had been measured prior to index traumas was significantly associated with PTSD severity following traumas.

Studies that measured neurobiological variables prospectively over at least two time points that were all following trauma exposure experiences were not included in this review. Studies that did not include measures of neurobiology prior to trauma experiences cannot causally address the DST. In addition, these types of studies have methodological limitations for addressing the NST because no matter how brief the span of time between trauma exposure and research assessment, any consequences to functional properties could have already occurred in that span of time.

Because there were different indices of neurobiology in these studies, strength of evidence for specific brain structures or peripheral variables was not reviewed. The purpose of this review is to survey the literature qualitatively for evidence in favor of either the DST or NST, and studies were characterized in regard to tests of any neurobiological variable supporting DST or NST in a study.

The analysis of studies is a qualitative review because the question was whether evidence amounts sufficiently either for or against two theories at a preliminary level and the variety of neurobiological systems included may involve different causal factors and marked methodological differences. Previous reviews have criticized systematic meta‐analyses for grouping different studies which may lead to meaningless estimates of effects and obscure discrepancies between different neurobiological systems (Eysenck, 1994).

## 3 RESULTS

**FIGURE 1:**
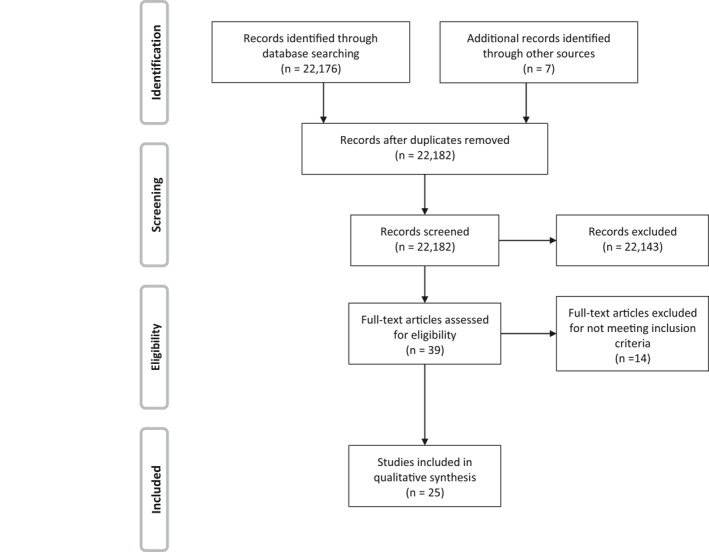
Preferred Items for Systemic Reviews and Meta‐Analysis flow diagram (n refers to number of studies)

The search returned 22,176 results, of which 17 studies met the inclusion criteria (Figure 1). Six of the eight studies in the DiGangi et al. review were found with this search method. Eleven new studies were found, including one study that was published before but not included in DiGangi et al.‘s review (Admon et al., 2009). Seven additional studies were found by reference searching (two studies from DiGangi et al. and five studies published since that review) and one additional study was provided by an anonymous reviewer. No studies published prior to 2005 met the inclusion criteria. A total of 25 studies met the inclusion criteria (Table 1).

**TABLE 1:** Twenty‐five second‐wave research studies of neurobiology and PTSD

	Sample	Re‐assessment timeline	Symptom severity following trauma	Supports DST or NST	Results
Brain imaging					
Admon et al., 2009	50 military paramedic recruits and 12 non‐recruit controls; 50% male	18 months	No subjects met criteria for PTSD.	Yes DST, Yes NST	DST: Greater amygdala activation on fMRI‐predicted post‐trauma symptoms. (Negative for hippocampus activation.) NST: Increased activation of hippocampus and NAcc in fMRI over time correlated with increased symptoms. (Negative for amygdala and subcallosal gyrus.)
van Wingen et al., 2011; van Wingen et al., 2013	32 soldiers pre‐deployment and 25 non‐deployed soldiers; 95% male	1.5 months after deployed. Re‐assessed 1.5 years later.	PTSD symptoms did not increase post‐deployment	No DST, No NST	DST: Amygdala and insula reactivity to negative emotion faces in fMRI. NST: Amygdala and insula reactivity in fMRI increased after 1.5 months, but no longer significant after 1.5 years.
Admon, Leykin, et al., 2013	A subset of Admon et al. (2009, p. 33) military paramedic recruits; 55% male	18 months	Only four subjects scored above PTSD cutoff	No DST, No NST	DST: Left hippocampus MRI. NST: They dichotomized the group into decreased and increased volume, which precluded a continuous test of degree of volume change in relation to PTSS severity.
Admon, Lubin, et al., 2013	24 military paramedic recruits; 50% male	18 months	Only four subjects developed mild PTSD severity	Yes DST	Greater amygdala activation on fMRI predicted PTSD symptoms. (Negative for NAcc and five other regions.)
Sekiguchi et al., 2013	42 college students exposed to earthquake; 79% male	Mean 101.8 days	No subjects met criteria for PTSD.	Yes DST, Yes NST	DST: Smaller right ventral ACC on MRI predicted PTSD symptoms. (Negative for amygdala, hippocampus, and insula.). NST: Reduction in size of left OFC in MRI associated with increased PTSD symptoms. (Negative for amygdala, hippocampus, and insula.)
McLaughlin et al., 2014	15 adolescents exposed to terrorist attack; 34% male	1 month	20% scored above PTSD cutoff	Yes DST	Greater left amygdala activation on fMRI in emotional reactivity task predicted more PTSS. Less hippocampal activation in emotion regulation task predicted more PTSS. (Negative for right amygdala and vmPFC in emotional reactivity task.)
HPA axis stress response					
Heinrichs et al., 2005	43 male firefighters	6, 9, 12, and 24 months after starting duty.	At 24 months, 16% met PTSD cutoff and 19% met subsyndromal PTSD cutoff.	No DST, No NST	DST: (Negative for (1) awakening cortisol plus 30, 45, and 60 min thereafter; (2) diurnal cortisol at four intervals; and (3) 24‐h epinephrine and norepinephrine). NST: (Negative for same three variables as above.)
Inslicht et al., 2011	263 police recruits; 86% male	12, 24, and 36 after starting duty.	At 36 months, 2% met PTSD cutoff.	No DST	(Greater cortisol awakening response predicted ASD but not PTSD.)
van Zuiden et al., 2011	34 male soldiers with PTSD, and 34 matched controls	1 and 6 months after deployed	Mean PTSS scores were moderately high.	Yes DST	Higher GR number on peripheral blood mononuclear cells predicted PTSD. (Negative for morning cortisol, mRNA expression in mononuclear cells, and leukocyte subsets.)
van Zuiden et al., 2012	448 male soldiers	6 months after deployed	At 6 months, 35 met PTSD cutoff.	Yes DST	Higher GR number on peripheral blood mononuclear cells, higher G1LZ mRNA and lower FKBP5 mRNA expression predicted PTSD. (Negative for morning cortisol, and SGK1 mRNA expression.)
Galatzer‐Levy et al., 2014	234 police recruits; 91% male	12, 24, 36, and 48 months after starting duty	Percent above cutoff not reported.	Yes DST	Lower cortisol response to lab stressor predicted membership in the reactive‐worsening class of PTSS.
van Zuiden et al., 2015	721 soldiers; 91% male	6 months after deployed	20% met PTSD cutoff	Yes DST	Greater T‐cell dexamethasone sensitivity predicted PTSD symptoms.
Steudte‐Schmiedgen et al., 2015	90 soldiers; 100% male	12 months after deployment	Percent above cutoff not reported.	Yes DST, No NST	DST: Lower baseline hair cortisol and lower cortisol responses to Trier Social Stress Test predicted PTSD symptoms. NST: (Negative for changes in hair cortisol.)
Autonomic stress response					
Guthrie & Bryant, 2005	87 male firefighters	2–28 days after an event	No subjects met criteria for acute stress disorder or PTSD.	Yes DST, No NST	DST: Greater startle response and greater SC to loud tones predicted PTSD. NST: (Negative for startle response and SC to loud tones.)
Guthrie & Bryant, 2006	87 male firefighters	24 months after starting duty.	No subjects met criteria for acute stress disorder or PTSD.	Yes DST	Reduced extinction of conditioned startle response predicted post‐trauma PTSD symptoms. (Negative for SC extinction.)
Pole et al., 2009	138 police recruits; 87% male	12 months after starting duty	One subject met full PTSD criteria and three met partial PTSD cutoff.	Yes DST	Greater startle response under medium threat, greater SC under low or high threat, and SC habituation slope predicted PTSD symptoms. (Negative for heart rate responses.)
Orr et al., 2012	99 trauma‐exposed firefighter, EMT, and police recruits; 91.8% male	Mean 12.3 months	No subject met cutoff for PTSD.	Yes DST	Greater EMG startle responses during extinction to loud tone phase predicted higher PTSS. (Negative for SC response during extinction; EMG, SC, and HR during acquisition phase.)
Busso et al., 2014	44 adolescents exposed to terrorist attack; 35% male	1 month	Percent above a cutoff not reported.	Yes DST	Low PEP reactivity plus high media exposure during Trier Social Stress Test, and high PEP reactivity plus low media exposure predicted higher PTSS. (Negative for RSA.)
Minassian et al., 2015	2160 male soldiers	6 months after deployed	4% met criteria for PTSD.	Yes DST	High LF:HF ratio of HRV at rest predicted PTSD status. (Negative for LF or HF alone.)
Pyne et al., 2016	343 Army national Guard soldiers	3 and 12 months after deployed	22% scored >34 on PCL at 3‐months post deployment.	Yes DST	Higher pre‐deployment PCL scores interacted with lower HF of HRV at rest to predict greater PTSS.
Mikolajewski & Scheeringa, 2016	36 four‐ to nine‐year old children exposed to Hurricane Katrina; 63·9% male	Mean 16 months	Mean 6.0 PTSD symptoms	Yes DST, No NST	DST: Lower resting RSA, and greater RSA reactivity predicted greater PTSS. NST: (Negative for resting RSA, and RSA reactivity)
Molecular					
Apfel et al., 2011	349 police recruits; 86% male	12 months	Mean 20.0 on PCL‐S	Yes DST	Elevated salivary MHPG during recovery phase after lab stressor predicted greater PTSS.
Glatt et al., 2013	24 male soldiers with PTSD and 24 matched controls	3 or 6 months after deployed	50% met PTSD cutoff by design	Yes DST	In transcriptome‐wide mRNA expression, 67 dysregulated probes (39 downregulated and 28 upregulated) predicted PTSD status.
Boks et al., 2016	92 male soldiers	6 months after deployed	One group scored above PTSD cutoff; two groups scored below	No DST, Yes NST	DST: (Negative for methylation of SKA2 gene.) NST: Lower methylation of SKA2 predicted greater PTSS.
Schür et al., 2017	92 male soldiers	6 months after deployed	One‐third met cutoff for PTSD by design.	No DST, No NST	DST: (Negative for glucocorticoid receptor exon 1_F_ methylation: (1) total methylation; (2) number of loci; (3) functional methylation.) NST: (Negative for same three variables as above.)

Abbreviations. ACC, anterior cingulate cortex; ASD, acute stress disorder; DST, diathesis stress theory; EMG, electromyogram; EMT, emergency medical technician; fMRI, functional magnetic resonance imaging; GR, glucocorticoid receptor; HF, high frequency; HRV, heart rate variability; LF, low frequency; MHPG, 3‐methoxy‐4‐hydroxyphenylglycol; NAcc, nucleus accumbens; NST, neurotoxic stress theory; OFC, orbitofrontal cortex; PCL, posttraumatic stress disorder checklist; PEP, pre‐ejection period; PTSD, posttraumatic stress disorder; PTSS, posttraumatic stress symptoms; RSA, respiratory sinus arrhythmia; SC, skin conductance; SKA2, spindle and kinetochore‐associated protein 2; vmPFC, ventromedial prefrontal cortex.

The 25 studies involved a total of 5675 participants. Neurobiological measures of each study are listed in Table 1. Sample sizes ranged from 15 to 2160. Twenty‐one studies involved young adults in the military, police, and firefighting. One study involved college students, two involved adolescents, and one involved children. The 15 largest studies, comprising 93% of all participants across all 25 studies, were 86%–100% male. Five studies included approximately 50% or more females. Details of each study are in Table 1.

### 3.1 Brain imaging

Of the six studies that used brain imaging, four supported the DST in relation to one or more brain structures (Admon et al., 2009; Admon, Lubin, et al., 2013; McLaughlin et al., 2014; Sekiguchi et al., 2013) and two did not (Admon, Leykin, et al., 2013; van Wingen, Geuze, Vermetten, & Fernández, 2011). Four of the six studies repeated imaging after index traumas and could test the NST; two of these supported the NST in relation to one or more brain structures (Admon et al., 2009; Sekiguchi et al., 2013) and two did not (Admon, Leykin, et al., 2013; van Wingen et al., 2011; van Wingen, Geuze, Vermetten, & Fernández, 2013).

### 3.2 HPA‐axis stress response systems

Of the seven studies that measured indices of the HPA system, five supported the DST in relation to one or more variables (Galatzer‐Levy et al., 2014; Steudte‐Schmiedgen et al., 2015; van Zuiden et al., 2011, 2012, 2015) and two did not (Heinrichs et al., 2005; Inslicht et al., 2011). Three of the seven studies also repeated the measurement of an HPA axis variable after the index trauma and were capable of testing the NST, and all were negative (Heinrichs et al., 2005; Steudte‐Schmiedgen et al., 2015; van Zuiden et al., 2015).

### 3.3 Autonomic stress response systems

Of the eight studies that measured indices of the autonomic nervous system (ANS), all eight supported the DST in relation to one or more variables (Busso, McLaughlin, & Sheridan, 2014; Guthrie & Bryant, 2005, 2006; Mikolajewski & Scheeringa, 2016; Minassian et al., 2015; Orr et al., 2012; Pole et al., 2009; Pyne et al., 2016). Two of the studies also repeated the measurement of an ANS variable after the index trauma and were capable of testing the NST, and both were negative for the NST (Guthrie & Bryant, 2005; Mikolajewski & Scheeringa, 2016).

### 3.4 Molecular

Of the four studies that measured molecular indices, two supported the DST in relation to one or more variables (Apfel et al., 2011; Glatt et al., 2013) and two did not (Boks et al., 2016; Schür et al., 2017). Two of the studies also repeated the measurement of a molecular variable after the index trauma; one supported the NST (Boks et al., 2016) and one failed to support the NST (Schür et al., 2017).

## 4 DISCUSSION

Of the 25 studies capable of testing DST, 19 were positive and 6 were negative. Of the 10 studies that tested the NST, 3 were positive and 7 were negative. The central premise of the NST – that psychological stress alters the brain, alters anatomical brain structures, and permanently disrupts hard‐wired neurocircuitry that has evolved through centuries of human development – is an extraordinary claim. As Carl Sagan said, “Extraordinary claims require extraordinary evidence” (Sagan, 1980). Extraordinary evidence in humans to support the NST premise in relation to PTSD appears lacking, at least in terms of prospective studies in humans.

Two of the three studies that were positive for the NST were brain imaging studies. One of these studies was a functional magnetic resonance imaging (fMRI) study that was positive for hippocampal and nucleus accumbens (NAcc) activation, but negative for amygdala activation (Admon et al., 2009). The other study was a volumetric MRI study that was positive for size reduction of the left orbitofrontal cortex (OFC), but negative for amygdala, hippocampus, and insula (Sekiguchi et al., 2013). It is noteworthy that several of the hallmarks of the NST model – size reductions in the hippocampus and amygdala, and amygdala reactivity – have no supporting studies in this second wave of neurobiology research.

The limited evidence for the NST contrasts with the rapid pace at which the NST has been embraced. Nearly all national agencies concerned with the well‐being of children and/or trauma have public‐facing statements which unequivocally claim that trauma causes neural alterations in the brain with accompanying psychiatric problems such as PTSD (American Academy of Pediatrics, 2019; National Child Traumatic Stress Network, 2019; Substance Abuse and Mental Health Services Administration, 2019). The claim that the NST is proven in relation to PTSD has also become a cornerstone of policy initiatives. In 2013, the Wisconsin senate became the first legislative body in the United States to pass a resolution which formally endorsed the NST model and resolved that all relevant future legislation take this into account. The resolution stated that stress and trauma exposure can permanently “shape the physical architecture of a child's developing brain and establish either a sturdy or a fragile foundation for all the learning, health, and behavior that follows” (Wisconsin State Senate, 2013). Similar resolutions have been adopted by city governments of Philadelphia (Beidas et al., 2013), Baltimore (Baltimore City Health Department, 2015), and New Orleans (Resolution No. R‐18‐344, 2018), and by both the United States Senate (U.S. Senate, 2018) and House of Representatives (U.S. House of Representatives, 2018). A more traditional, conservative, and rational approach would be to wait until a theory is replicated by studies with appropriately causal designs (e.g., prospective longitudinal) before promoting it as true.

While it may be considered prudent to err on the side of safety and do everything possible to protect individuals from psychological trauma, this promotion of the NST raises two concerns. The first concern is that many stakeholders appear to have a misunderstanding of the extant evidence base. The second‐wave studies appear to be unknown to researchers and journalists who assert unequivocally that trauma permanently alters the brain. Belief in the NST appears to be based largely on the first wave of cross‐sectional studies, even though it is well known that cross‐sectional studies have little to no causal explanatory power because they cannot determine the direction of the relationships between variables.

### 4.1 The property of exchangeability in cross‐sectional studies and trauma research

It is possible that premature support for the NST in relation to PTSD comes from a failure to recognize that a flaw of cross‐sectional trauma studies is that they violate the property of exchangeability. Cross‐sectional studies are case–control studies in which individuals with the outcome of PTSD/high PTSS are the cases, and individuals with no PTSD/low PTSS are the controls. The principle of exchangeability states that the groups of cases and controls need to be equal on all salient variables prior to their exposures to the variable of interest. Prior to exposure experiences, if a member of one group was swapped into the other group, it would have no effect on group means of the other variables at the beginning of the study. In other words, prior to naturalistic exposures, members were exchangeable between groups. As Rothman and Greenland's basic text on epidemiology noted, “Controls should be selected from the same population – the source population or study base – that gives rise to the cases” (Rothman & Greenland, 1998, p. 97).

Several studies have illustrated how the property of exchangeability can be violated in trauma research. In Nilsson, Gustafsson, and Svedin's (2012) study of 462 adolescents in Sweden, cumulative traumatic experiences were strongly positively correlated with adverse family circumstances (e.g., divorce, a parent who spent time in jail, or a parent with problems of alcohol or other drugs), indicating that trauma does not happen at random; trauma occurs more frequently to children in families with other adverse circumstances. The population of adolescents with cumulative trauma experiences was not exchangeable on variables of family circumstances with the population of adolescents with fewer trauma experiences (Nilsson et al., 2012).

In Scheeringa's (2015) study of 284 very young children (3–6 years old), children who experienced repeated trauma had higher rates of oppositional defiant disorder *prior* to their first traumas compared to children who experienced single‐event trauma. Before children experienced their first traumas, those who would eventually experience repeated trauma had different psychiatric profiles and were not exchangeable with children who would experience only one trauma (Scheeringa, 2015).

Danese and colleagues examined over 3000 adults in two cohorts that had been followed from birth. Impairments in cognitive functions (e.g., general intelligence, executive function, processing speed, memory, perceptual reasoning, and verbal comprehension) *predated* childhood victimization (Danese et al., 2017). Prior to experiences of childhood victimization, children who would later be victimized were not exchangeable with children who would never be victimized on a wide array of cognitive functions.

When the property of exchangeability is violated, it could mean there are one or more hidden confounders. It is likely that whatever third variables cause adverse family circumstances (Nilsson et al., 2012), oppositional defiant disorder (Scheeringa, 2015), and cognitive impairments (Danese et al., 2017) are hidden confounding variables that are also related to trauma exposure. This violation of exchangeability may lead researchers to mistakenly infer that differences in neurobiology postdated trauma exposure when they actually predated trauma exposure.

### 4.2 Trauma research and social policy

The second concern is how research has become associated with social policy. Prior to 2010, mention of the NST was constrained to thought‐provoking titles of papers until several notable events had a cumulative effect. The first event was publication of the first Adverse Childhood Experiences (ACE) study in 1998 (Felitti et al., 1998), followed by approximately 20 studies by the same research group over the next 10 years with a consistent conclusion that childhood adverse events *caused* a wide range of mental and physical ailments. The types of experiences under the ACE umbrella include true trauma, but also include stressful experiences that are not true trauma experiences. Nevertheless, the popularity of the ACE conclusion gave generalizability of the NST concept beyond psychiatry.

Concurrently in 2000, pediatrician Jack Shonkoff authored a major policy review for the National Academy of Sciences, titled *From Neurons to Neighborhoods*, in which he coined the concept of “stressors that are toxic” (National Research Council and Institute of Medicine, 2000). In 2003, Shonkoff spearheaded the creation of the National Scientific Council on the Developing Child, which despite its name is not a national agency. The Council is a private group of scientists and stakeholders in early childhood with an administrative home at Harvard University, with a stated mission to close the “gap between science and policy” (Center on the Developing Child at Harvard University, 2014, p. 3). The Council members agreed to market the phrase “toxic stress” to convey their message to the public because, by their own admission, “Just saying ‘stress’ more loudly wasn't going to get them where they needed to go” (p. 8). The Council produced a report in 2005 that coined the phrase “toxic stress,” and has pursued a wide‐ranging public communication and legislative agenda (National Scientific Council on the Developing Child, 2005). The Council appears to have been successful in their mission. We conducted a literature search in PsychInfo and PubMed for the term “toxic stress” and related phrases in peer‐reviewed publications; the term appeared only twice prior to 2010 but has appeared 88 times in titles or abstracts since 2010.

This was followed by the publication in 2014 of the book *The Body Keeps the Score*, by trauma expert Bessel van der Kolk, which became the number one best‐selling book, not just in the categories of psychological trauma and PTSD, but in all of psychiatry to the present day (amazon.com, 2019). The book by van der Kolk, which is often cited for popularizing the NST concept, relies on first‐wave research. The cumulative effect of these events, based on interpretations from the first wave of research, appears to have struck a chord and created a favorable view of the NST model that has influenced our professional and governmental agencies and spread into social policy changes.

### 4.3 Directions for future research

The extant body of second‐wave studies is overly represented by samples of male adults self‐selected for dangerous professions who may be disproportionately resilient. Future prospective pre‐trauma studies of naturalistic opportunities that are not limited to first‐responder professions are more likely to avoid violating the principle of exchangeability. More samples of young children are needed to examine the notion of developmental periods of vulnerability. Prevention and treatment research may need to take the DST into consideration. For example, programs to prevent PTSD in first responders are unlikely to help those with the diathesis to develop PTSD. By recognizing a diathesis, those who possess it may either be guided to careers that do not involve routine exposure to life‐threatening situations or to try to build resilience.

### 4.4 Limitations

Sample sizes of several studies in this review were small, and thus any bias within small studies could skew the findings toward either theory. Only one study in this review involved children and two involved adolescents. As many supporters of the NST have already concluded that early childhood is a period of unique vulnerability, additional pre‐trauma, prospective studies in youths are needed. The majority of participants in pre‐trauma studies have been male. If a permanent effect consistent with the NST was operable only in females, these studies may not have found it. However, proponents of the NST have never asserted that it is a gender‐specific effect.

Many of the subjects in these studies experienced traumas prior to their index traumas in the studies. However, two studies included samples with no prior trauma exposure and both found support for the DST (Admon, Lubin, et al., 2013; Mikolajewski & Scheeringa, 2016). The majority of participants in the studies were self‐selected individuals who entered military and first‐responder careers. These individuals may be relatively more resilient to the effects of stress. However, if they developed PTSD at lower rates than more vulnerable individuals, this may have made it more difficult to find evidence in favor of both theories. It is noteworthy that if these subjects were inherently resilient, that is an argument in favor of the DST. The pre‐trauma, prospective design of these studies does not mean that they were automatically free of confounding. Those who were exposed to index traumas, or exposed more frequently, may have been different from those non‐exposed or less exposed during the duration of the studies due to hidden variables. For example, they may have been more impulsive, braver, or less self‐protective which led them to be drawn toward life‐threatening situations.

An argument may be made that the effect of trauma may be different in situations of prolonged, repeated, severe, and/or interpersonal trauma, such as cases of sexual abuse, torture, and domestic violence (Herman, 1992), and those types of experiences were not in the extant pre‐trauma prospective studies. Until pre‐trauma prospective studies are conducted with those populations, it is impossible to address that concern with evidence. It does however make the point that such data with those populations do not exist either for or against the NST.

An argument may be made that the studies in this review examined the wrong developmental period by claiming that neurobiological differences that appear to be an inherent diathesis were actually caused by stress in the womb, consistent with the NST. Indeed, there is already another group of researchers making this case. Yet, the latest review on this subject made clear how little evidence exists in humans to support this speculation (O'Donnell & Meaney, 2017).

## 5 CONCLUSION

This qualitative review indicates that a consensus supporting the NST was initially drawn during the first wave of research when the only studies that existed in humans were cross‐sectional studies, and the power of cross‐sectional studies to determine causality is low. This review suggests that closure around the NST model is premature. The NST has no precedent as a model of dysfunction in the rest of medicine, and its popularity appears to be in part due to advocacy. Some victims of trauma have begun to push back against being defined by the NST because they do not view themselves as permanently altered goods (O'Connor, 2019). An advocacy message that was meant to help them may actually negatively label them. How the trauma field responds to these issues in the future has implications for how we understand the very nature of the hardiness versus the fragility of the human mind, and may have widespread policy ramifications.

## CONFLICT OF INTEREST

The author declares no conflicts of interest.
